# Supplementary figures and images for: IMD-mediated innate immune priming increases Drosophila survival and reduces pathogen transmission
Source: PLoS Pathog. 2024 Jun 10;20(6):e1012308. doi: 10.1371/journal.ppat.1012308 (PMC11192365; doi:10.1371/journal.ppat.1012308)

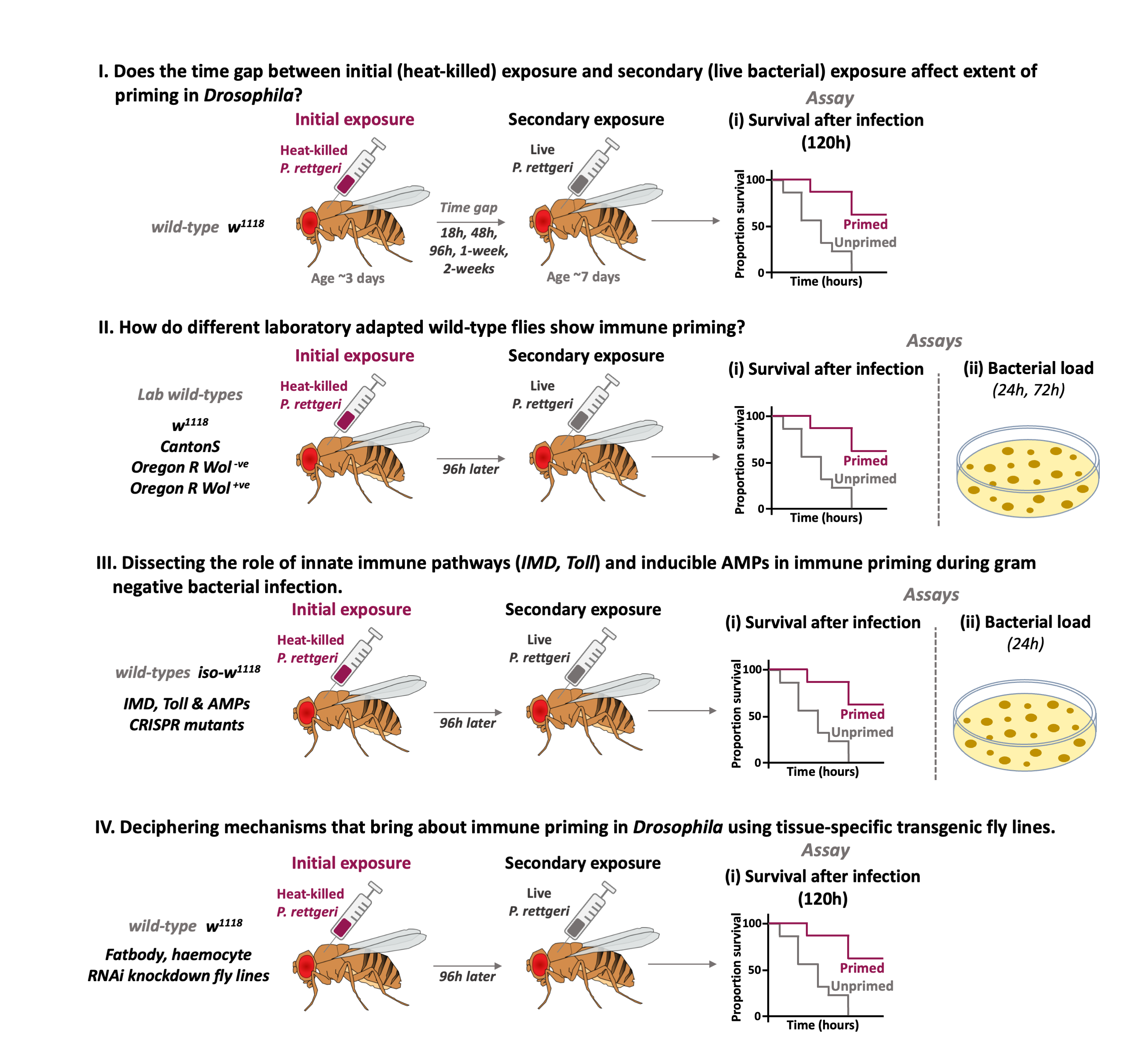

Supplement: S1 Fig — Schematic representation of different priming experiments aimed at (I). testing whether the length of the period between primary heat-killed exposure and the secondary pathogenic challenge affects the extent of priming (II). how different lab-adapted control/genetic background flies vary in priming (III). dissecting the role of innate immune pathways (IMD and Toll) and inducible AMPs in immune priming and (IV). deciphering mechanisms that bring about immune priming in Drosophila using tissue-specific fat body and haemocytes UASRNAi mutants. The experimental design for priming assays includes survival and internal bacterial load quantification. (TIFF) [file ppat.1012308.s002.tiff]

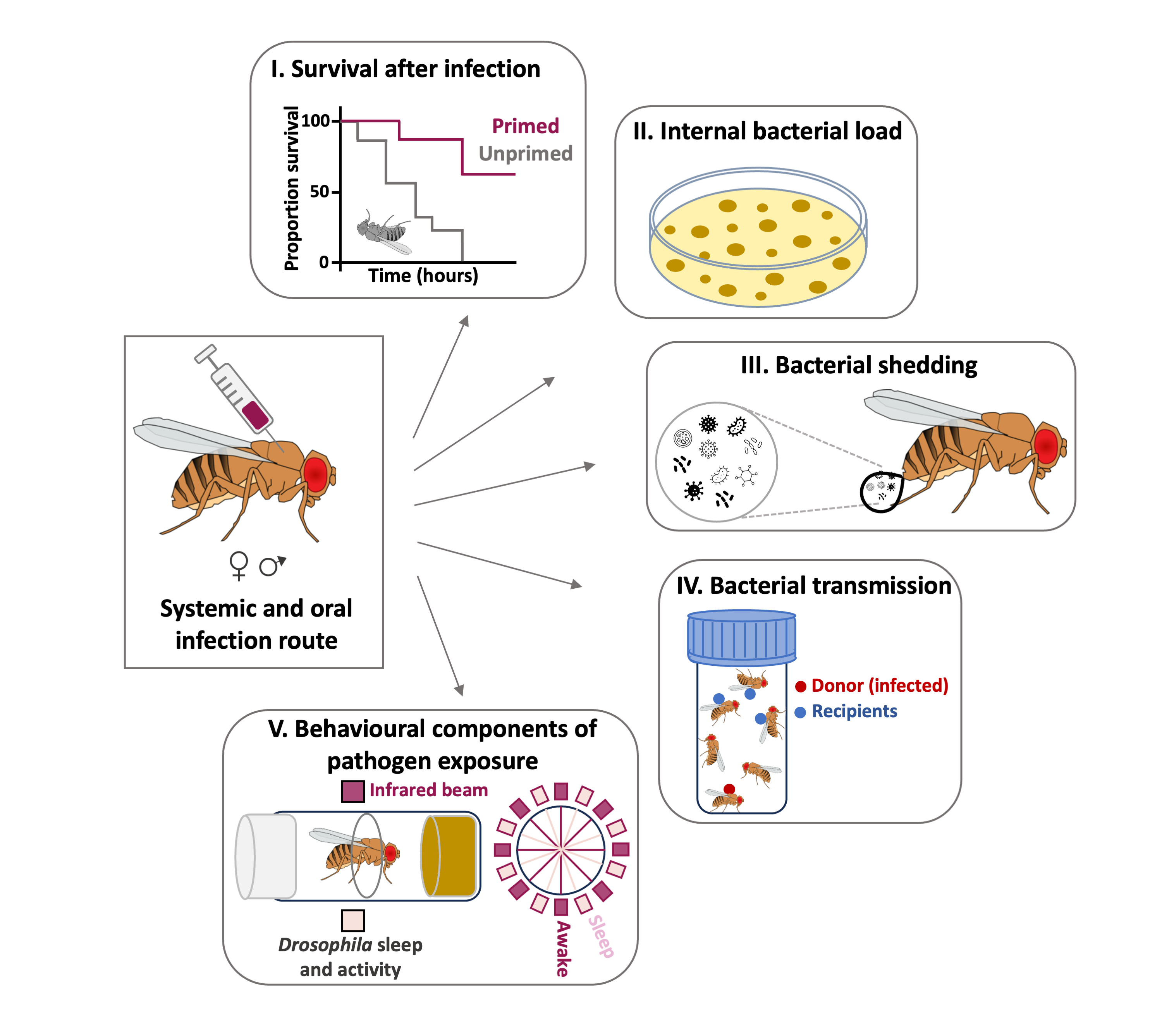

Supplement: S2 Fig — The assays include (I). survival following different infection routes (II). internal bacterial load (III). behavioural components of pathogen exposure such as sleep and awake activity (IV). bacteria shedding and (V). transmission. n = 6–7 vials of 8–12 flies in each vial, for each treatment and sex combination. (TIFF) [file ppat.1012308.s003.tiff]

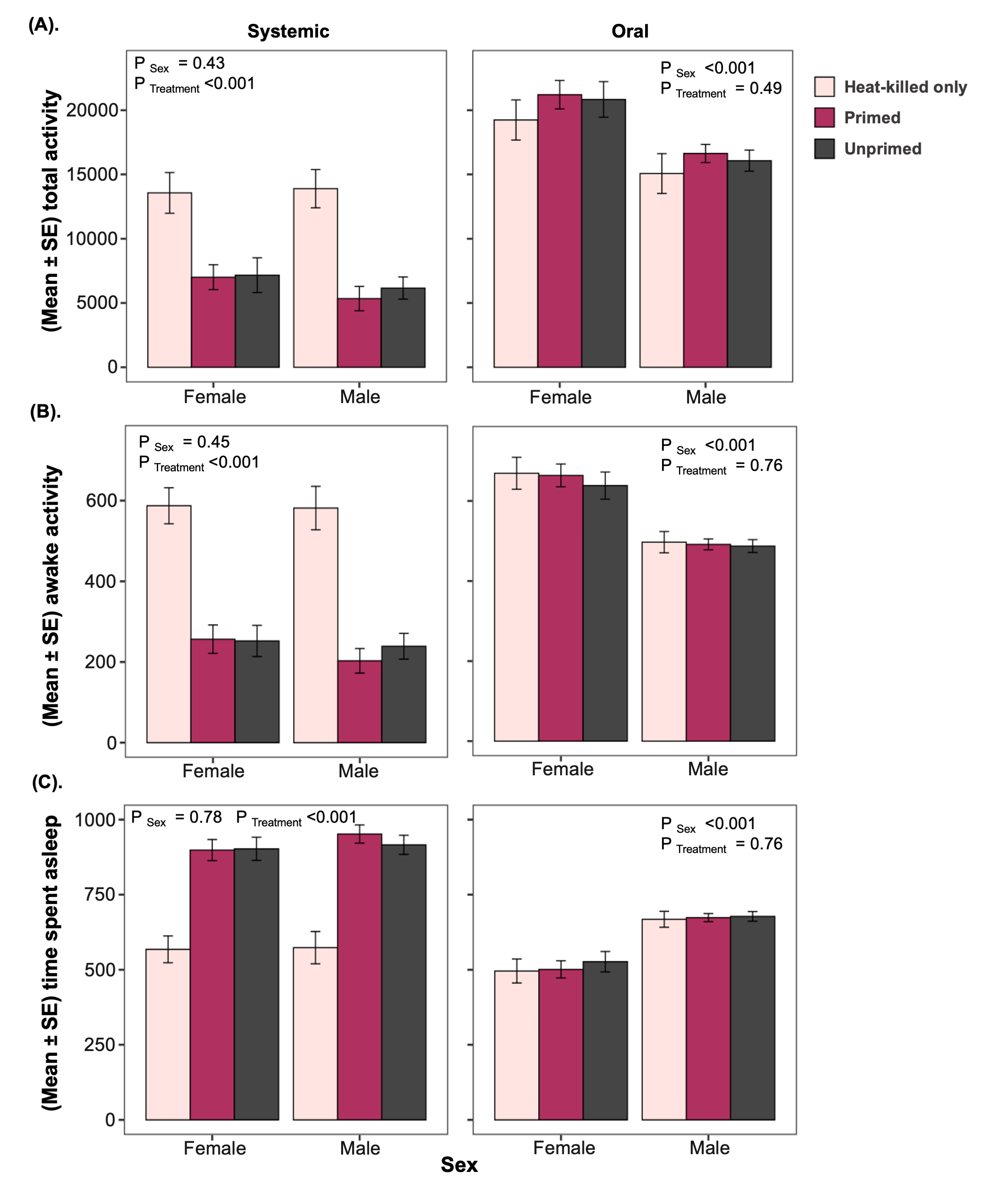

Supplement: S3 Fig — Mean ±SE total locomotor activity for males and females (n = 52 individual flies per treatment), during first 72-hours following systemic and oral priming and infection. (A) average total locomotor activity (B) average awake activity and (C) proportion of flies spent sleeping. (TIFF) [file ppat.1012308.s004.tiff]

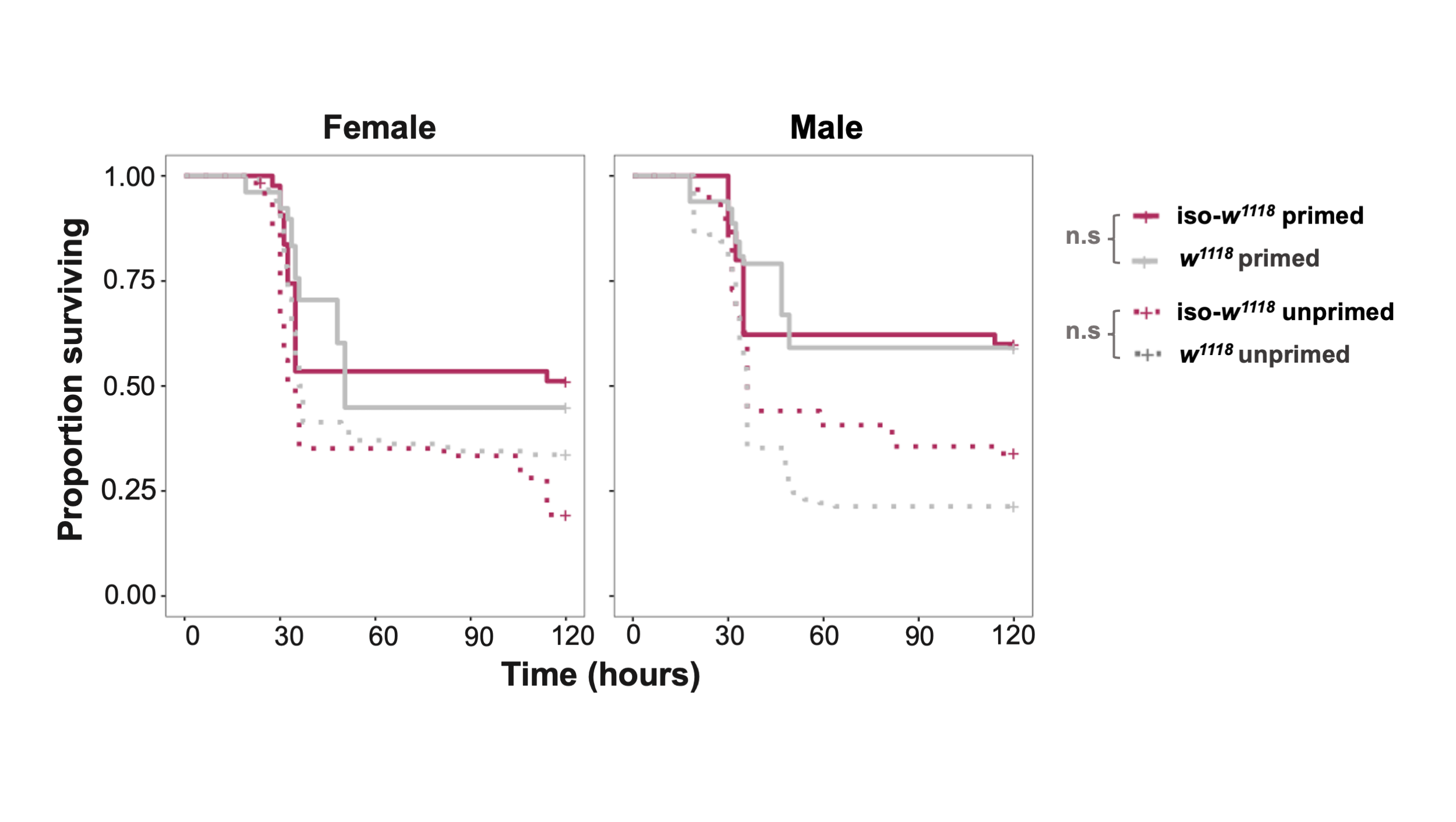

Supplement: S4 Fig — flies after initial heat-killed exposure and followed by live P. rettgeri infection with OD600 = 0.1. As another control, we infected both w1118 and iso-w1118 control because the CRISPR/cas9 AMP mutants we used were on the iso-w1118 background, so we wanted to confirm that any changes in priming were not due to the background of the mutants, as opposed to the mutations. We found that the differences between w1118 and iso-w1118 (primed and unprimed treatments) were not significantly different. (TIFF) [file ppat.1012308.s005.tiff]

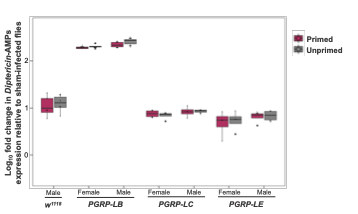

Supplement: S5 Fig — 24-hours after exposure to live P. rettgeri in male and female control w1118 flies and flies with loss-of-function in different PGRPs, PGRP-LB, PGRP-LC & -LE. (TIFF) [file ppat.1012308.s006.tiff]
