# Supplementary material for: IMD-mediated innate immune priming increases Drosophila survival and reduces pathogen transmission
Source: PLoS Pathog. 2024 Jun 10;20(6):e1012308. doi: 10.1371/journal.ppat.1012308 (PMC11192365; doi:10.1371/journal.ppat.1012308)
Supplement: S1 Table — (DOCX) [file ppat.1012308.s007.docx]

S1 Table. Summary of mixed effects Cox model, fitting the model to estimate time-delayed priming response in control w1118 male and female flies. We used data from individuals exposed to live bacterial after different time intervals of initial heat-killed *P. rettgeri* exposure. We specified the model as: survival ~ treatment x sex x timepoint (1|vial/block), with treatment and sex as fixed effects, and vials nested within each block and as a random effect. The table shows model output (ANOVA) for priming in control flies.

| **Timepoint/sex** | **Source** | **loglik** | **χ2** | **Df** | **P** |
| --- | --- | --- | --- | --- | --- |
| ***18-hours*** *post*  *Priming overall* | Treatment  Sex  Sex × Treatment | -801.96  -790.74 | 15.92  22.44 | 1  1 | **<0.001**  **<0.001** |
|  |  | - 781.47 | 18.54 | 1 | **<0.001** |
|  | *Random effects*  *Vials/block* | *Std Dev* |  |  |  |
|  |  | *0.08* |  |  |  |
| *Female* | Treatment | -433.36 | 0.64 | 1 | 0.42 |
|  | *Random effects*  *Vials/block* | *Std Dev*  *0.18* |  |  |  |
| *Male* | Treatment | -247.71 | 32.37 | 1 | **<0.001** |
|  | *Random effects* | *Std Dev* |  |  |  |
|  | *Vials/block* | *0.009* |  |  |  |
| ***48-hours*** *post*  *priming* | Treatment  Sex  Sex × Treatment | -1050.2  - 1045.7 | 25.73  8.94 | 1  1 | **<0.001**  **0.002** |
| *overall* |  | -1045.7 | 0.08 | 1 | 0.76 |
|  | *Random effects*  *Vials/block* | *Std Dev* |  |  |  |
|  |  | *0.004* |  |  |  |
| *Female* | Treatment | -315.12 | 21.15 | 1 | **<0.001** |
|  | *Random effects*  *Vials/block* | *Std Dev*  *0.23* |  |  |  |
| *Male* | Treatment | -239.08 | 15.63 | 1 | **<0.001** |
|  | *Random effects* | *Std Dev* |  |  |  |
|  | *Vials/block* | *0.15* |  |  |  |
| ***96-hours*** *post*  *Priming* | Treatment  Sex  Sex × Treatment | -647.58  -647.51 | 36.35  0.14 | 1  1 | **<0.001**  0.70 |
| *overall* |  | -647.50 | 0.006 | 1 | 0.93 |
|  | *Random effects* | *Std Dev* |  |  |  |
|  | *Vials/block* | 0.15 |  |  |  |
| *Female* | Treatment | -511.02 | 14.63 | 1 | **<0.001** |
|  | *Random effects* | *Std Dev* |  |  |  |
|  | *Vials/block* | *0.009* |  |  |  |
| *Male* | Treatment | -399.48 | 12.69 | 1 | **<0.001** |
|  | *Random effects*  *Vials/block* | *Std Dev* |  |  |  |
|  |  | *0.008* |  |  |  |
| ***168-hours*** *post*  *Priming*  *overall* | Treatment  Sex  Sex × Treatment | -949.67  -948.59  -946.20 | 11.712  2.1658  4.7705 | 1  1  1 | **0.0006**  0.14  **0.02** |
|  | *Random effects* | *Std Dev* |  |  |  |
|  | *Vials/block* | *0.009* |  |  |  |
| *Female* | Treatment | -448.02 | 1.1289 | 1 | 0.28 |
|  | *Random effects* | *Std Dev* |  |  |  |
|  | *Vials/block* | *0.009* |  |  |  |
| *Male* | Treatment | -371.76 | 13.381 | 1 | **<0.001** |
|  | *Random effects* | *Std Dev* |  |  |  |
|  | *Vials/block* | *0.009* |  |  |  |
| ***336-hours*** *post*  *Priming*  *overall* | Treatment  Sex  Sex × Treatment | -1035.0  -1034.9  -1033.5 | 5.3983  0.1854  2.7814 | 1  1  1 | **0.02**  0.66  0.09 |
|  | *Random effects* | *Std Dev* |  |  |  |
|  | *Vials/block* | *0.004* |  |  |  |
| *Female* | Treatment | -445.91 | 0.2168 | 1 | 0.64 |
|  | *Random effects* | *Std Dev* |  |  |  |
|  | *Vials/block* | *0.01* |  |  |  |
| *Male* | Treatment | -448.08 | 8.345 | 1 | **0.003** |
|  | *Random effects* | *Std Dev* |  |  |  |
|  | *Vials/block* | *0.008* |  |  |  |
