# Supplementary material for: IMD-mediated innate immune priming increases Drosophila survival and reduces pathogen transmission
Source: PLoS Pathog. 2024 Jun 10;20(6):e1012308. doi: 10.1371/journal.ppat.1012308 (PMC11192365; doi:10.1371/journal.ppat.1012308)
Supplement: S2 Table — (DOCX) [file ppat.1012308.s008.docx]

S2 Table. Summary of mixed effects Cox model, fitting the model to estimate priming response in different laboratory control w1118 male and female flies. We used data from the unprimed-infected and the primed-infected treatments and specified the model as: survival ~ treatment x sex x (1|vial/block), with treatment and sex as fixed effects, and vials nested within each block and as a random effect. The table shows model output (ANOVA) for priming in control flies.

| **Fly strain** | **Source** | **loglik** | **χ2** | **Df** | **P** |
| --- | --- | --- | --- | --- | --- |
| *w^1118^* | Treatment  Sex  Sex × Treatment | -647.58  -647.51 | 36.35  0.146 | 1  1 | **<0.001**  0.70 |
|  |  | -647.50 | 0.007 | 1 | 0.93 |
|  | *Random effects*  *Vials/block* | *Std Dev* |  |  |  |
|  |  | *0.15* |  |  |  |
| *Canton-S* | Treatment  Sex  Sex × Treatment | -633.00  -619.47 | 11.56  27.04 | 1  1 | **<0.001**  **<0.001** |
|  |  | -618.39 | 2.174 | 1 | 0.14 |
|  | *Random effects*  *Vials/block* | *Std Dev* |  |  |  |
|  |  | *0.008* |  |  |  |
| *OreR^Wol+^* | Treatment  Sex  Sex × Treatment | -436.34  -436.04 | 0.455  0.607 | 1  1 | 0.49  0.43 |
|  |  | -432.80 | 6.482 | 1 | **0.01** |
|  | *Random effects*  *Vials/block* | *Std Dev* |  |  |  |
|  |  | *0.17* |  |  |  |
| *OreR^Wol-^* | Treatment  Sex  Sex × Treatment | -610.99  -609.91 | 26.18  2.171 | 1  1 | **<0.001**  0.14 |
|  |  | -604.13 | 11.54 | 1 | **<0.001** |
|  | *Random effects*  *Vials/block* | *Std Dev* |  |  |  |
|  |  | *0.02* |  |  |  |
