# Supplementary material for: IMD-mediated innate immune priming increases Drosophila survival and reduces pathogen transmission
Source: PLoS Pathog. 2024 Jun 10;20(6):e1012308. doi: 10.1371/journal.ppat.1012308 (PMC11192365; doi:10.1371/journal.ppat.1012308)
Supplement: S3 Table — (DOCX) [file ppat.1012308.s009.docx]

S3 Table. Summary of mixed effects Cox model, fitting the model to estimate the impact Wolbachia on immune priming response using genetic background OreR male and female flies. We used data from the unprimed-infected and the primed-infected treatments and specified the model as: survival ~ treatment x sex x *Wolbachia* status (1|vial/block), with treatment and sex as fixed effects, and vials nested within each block and as a random effect. The table shows model output (ANOVA) for priming in different control/genetic background flies.

1. **Impact of *Wolbachia***

| **Fly strain** | **Source** | **loglik** | **χ2** | **Df** | **P** |
| --- | --- | --- | --- | --- | --- |
| *OreR* | Treatment  Sex  *Wolbachia* status  Sex × Treatment  Treatment x *Wol* status  Sex x *Wol* status  Sex x Treatment x *Wol* status | -1213.1  -1210.9  -1197.4 | 12.88  4.31  27.06 | 1  1  1 | **<0.001**  **0.037**  **<0.001** |
|  |  | -1189.5  -1183.8  -1183.4  -1183.4 | 15.84  11.36  0.69  0.06 | 1  1  1  1 | **<0.001**  **<0.001**  0.40  0.79 |
|  | *Random effects*  *Vials/block* | *Std Dev* |  |  |  |
|  |  | *0.004* |  |  |  |

1. ***Wolbachia* infection on males and females separately**

| **Sex** | **Source** | **loglik** | **χ2** | **Df** | **P** |
| --- | --- | --- | --- | --- | --- |
| *Female* | Treatment  *Wol* status  Treatment x *Wol* status | -514.76  -506.29  -503.72 | 0.10  16.94  5.13 | 1  1  1 | 0.74  **<0.001**  **0.02** |
|  | *Random effects* | *Std Dev* |  |  |  |
|  | *Vials/block* | 0.008 |  |  |  |
| *Male* | Treatment  *Wol* status  Treatment x *Wol* status | -535.18  -529.14  -525.88 | 23.90  12.07  6.52 | 1  1  1 | **<0.001**  **<0.001**  **0.01** |
|  | *Random effects* | *Std Dev* |  |  |  |
|  | *Vials/block* | 0.009 |  |  |  |
