# Supplementary material for: IMD-mediated innate immune priming increases Drosophila survival and reduces pathogen transmission
Source: PLoS Pathog. 2024 Jun 10;20(6):e1012308. doi: 10.1371/journal.ppat.1012308 (PMC11192365; doi:10.1371/journal.ppat.1012308)
Supplement: S4 Table — (DOCX) [file ppat.1012308.s010.docx]

S4 Table. Summary of mixed effects Cox model, fitting the model to estimate priming response in male and female control w1118 flies. We used data from the unprimed-infected and the primed-infected treatments and specified the model as: survival ~ Treatment x sex x (1|vial/block), with treatment and sex as fixed effects, and vials within a block as a random effect. The table shows model output (ANOVA).

| **Fly strain** | **Source** | **loglik** | **χ2** | **Df** | **P** |
| --- | --- | --- | --- | --- | --- |
| *w^1118^* | Sex  Treatment  Sex × Treatment | -5144.0  -5163.8 | 39.56  0.351 | 1  1 | **<0.001**  **<0.001** |
|  |  | -5143.5 | 0.921 | 1 | 0.34 |
|  | *Random effects*  *Vials/block* | *Std Dev* |  |  |  |
|  |  | *0.453* |  |  |  |
