# Supplementary material for: IMD-mediated innate immune priming increases Drosophila survival and reduces pathogen transmission
Source: PLoS Pathog. 2024 Jun 10;20(6):e1012308. doi: 10.1371/journal.ppat.1012308 (PMC11192365; doi:10.1371/journal.ppat.1012308)
Supplement: S5 Table — (DOCX) [file ppat.1012308.s011.docx]

S5 Table. Summary of log10 transformed bacterial load data after 0.2 OD *P. rettgeri* infection, analysed using a non-parametric test for ANOVA (Kruskal-Wallis test) by fitting ‘treatment’, as fixed-effects for female and male control *w^1118^.*

| **Time** | **Sex** | **Source** | **Chi Sq.** | **Df** | **P** |
| --- | --- | --- | --- | --- | --- |
| ***24-hours*** *following infection* | *Female*  *Male* | Treatment  Treatment | 3.2495  8.3974 | 1  1 | 0.07  **0.003** |
| ***72-hours*** *following infection* | *Female*  *Male* | Treatment  Treatment | 0.2102  1.8293 | 1  1 | 0.64  0.17 |
