# Supplementary material for: IMD-mediated innate immune priming increases Drosophila survival and reduces pathogen transmission
Source: PLoS Pathog. 2024 Jun 10;20(6):e1012308. doi: 10.1371/journal.ppat.1012308 (PMC11192365; doi:10.1371/journal.ppat.1012308)
Supplement: S6 Table — (DOCX) [file ppat.1012308.s012.docx]

S6 Table: Summary of Cox prop-hazard model, for female and male flies of wild type w1118. We used data from 3 to 7-day adult male and females infected with OD_600_ = 0.75 dose for systemic and dose OD_600_ = 25 for oral priming and infection with *P. rettegeri*. We specified the models as survival ~ sex x treatment with ‘treatment’, and ‘sex’ as fixed effects. Table shows model output (ANOVA) for survival post-infection for male and female *w^1118^* flies.

| ***Response*** | ***Predictor*** | ***df*** | ***Chi sq*** | ***p*** |
| --- | --- | --- | --- | --- |
| **Systemic Infection route** | Sex | 1 | 1.383 | 0.23 |
|  | Treatment | 2 | 100.7 | **<0.001** |
|  | Sex x Treatment | 2 | 3.103 | 0.21 |
| **Oral Infection route** | Sex | 1 | 0.728 | 0.39 |
|  | Treatment | 2 | 85.76 | **<0.001** |
|  | Sex x Treatment | 2 | 3.421 | 0.180 |
