# Supplementary material for: IMD-mediated innate immune priming increases Drosophila survival and reduces pathogen transmission
Source: PLoS Pathog. 2024 Jun 10;20(6):e1012308. doi: 10.1371/journal.ppat.1012308 (PMC11192365; doi:10.1371/journal.ppat.1012308)
Supplement: S7 Table — (DOCX) [file ppat.1012308.s013.docx]

S7 Table: Summary of log transformed bacterial load data after OD_600_=0.75 systemic and OD_600_=25 oral *P. rettgeri* systemic infection for male and female *w^1118^* flies analysed using a non-parametric Wilcoxon (Kruskal-Wallis) test by fitting ‘Sex’ as categorical fixed-effects after 24-hours post systemic and oral priming and infection.

| ***Response*** | ***Sex*** | ***Predictor*** | ***Chi sq*** | ***df*** | ***p*** |
| --- | --- | --- | --- | --- | --- |
| ***Systemic route*** | Female | Treatment | 0.403 | 1 | 0.52 |
|  | Male | Treatment | 6.090 | 1 | **0.01** |
| ***Oral route*** | Female | Treatment | 0.255 | 1 | 0.61 |
|  | Male | Treatment | 3.870 | 1 | **0.04** |
