# Supplementary material for: IMD-mediated innate immune priming increases Drosophila survival and reduces pathogen transmission
Source: PLoS Pathog. 2024 Jun 10;20(6):e1012308. doi: 10.1371/journal.ppat.1012308 (PMC11192365; doi:10.1371/journal.ppat.1012308)
Supplement: S8 Table — (DOCX) [file ppat.1012308.s014.docx]

S8 Table: Model outputs for statistical test (GLM) performed on host activity data, that is, locomotor activity, sleep patterns and average awake activity in males and females of *w^1118^* during systemic and oral priming and infection respectively.

| ***Infection*** | ***Response*** | ***Predictor*** | ***df*** | ***F ratio*** | ***p*** |
| --- | --- | --- | --- | --- | --- |
| ***Oral*** | *Total activity* | Sex | 1 | 16.82 | **<0.001** |
|  |  | Treatment | 2 | 0.711 | 0.49 |
|  |  | Sex x Treat | 2 | 0.020 | 0.97 |
|  | *Awake activity* | Sex | 1 | 40.86 | **<0.001** |
|  |  | Treatment | 2 | 0.269 | 0.76 |
|  |  | Sex x Treat | 2 | 0.112 | 0.89 |
|  | *Time asleep* | Sex | 1 | 40.86 | **<0.001** |
|  |  | Treatment | 2 | 0.269 | 0.76 |
|  |  | Sex x Treat | 2 | 0.112 | 0.89 |
| ***Systemic*** | *Total activity* | Sex | 1 | 0.612 | 0.43 |
|  |  | Treatment | 2 | 19.18 | **<0.001** |
|  |  | Sex x Treat | 2 | 0.294 | 0.74 |
|  | *Awake activity* | Sex | 1 | 0.555 | 0.45 |
|  |  | Treatment | 2 | 41.03 | **<0.001** |
|  |  | Sex x Treat | 2 | 0.238 | 0.78 |
|  | *Time asleep* | Sex | 1 | 0.555 | 0.45 |
|  |  | Treatment | 2 | 41.03 | **<0.001** |
|  |  | Sex x Treat | 2 | 0.238 | 0.78 |
