# Supplementary material for: IMD-mediated innate immune priming increases Drosophila survival and reduces pathogen transmission
Source: PLoS Pathog. 2024 Jun 10;20(6):e1012308. doi: 10.1371/journal.ppat.1012308 (PMC11192365; doi:10.1371/journal.ppat.1012308)
Supplement: S9 Table — (DOCX) [file ppat.1012308.s015.docx]

S9 Table: Summary of non-parametric Wilcoxon (Kruskal-Wallis) test for oral bacterial shedding (log transformed bacterial load) after 4-hours of oral priming and infection with *P. rettgeri* OD_600_=25. Bacterial transmission (percentage of recipient flies with measurable bacterial load) after 4-hours of exposure with infection (donor) *w^1118^* after oral priming and infection.

| ***Response*** | ***Sex*** | ***Predictor*** | ***Chi sq*** | ***df*** | ***p*** |
| --- | --- | --- | --- | --- | --- |
| ***Bacterial Shedding*** | Female | Treatment | 0.003 | 1 | 0.95 |
|  | Male | Treatment | 17.29 | 1 | **<0.001** |
| ***Transmission*** | Female | Treatment | 0.187 | 1 | 0.66 |
|  | Male | Treatment | 5.444 | 1 | **0.019** |
