# Supplementary material for: IMD-mediated innate immune priming increases Drosophila survival and reduces pathogen transmission
Source: PLoS Pathog. 2024 Jun 10;20(6):e1012308. doi: 10.1371/journal.ppat.1012308 (PMC11192365; doi:10.1371/journal.ppat.1012308)
Supplement: S10 Table — (DOCX) [file ppat.1012308.s016.docx]

S10 Table. Summary of mixed effects Cox model, fitting the model to estimate priming response in male and female control w1118, IMD and Toll transgenic flies. We used data from the unprimed-infected and the primed-infected treatments and specified the model as: survival ~ Treatment x sex x (1|vial/block), with treatment and sex as fixed effects, and vials within a block as a random effect for each fly line. The table shows model output (ANOVA).

| **Fly strain** | **Source** | **loglik** | **χ2** | **Df** | **P** |
| --- | --- | --- | --- | --- | --- |
| *iso w^1118^* | Sex  Treatment  Sex × Treatment | -1597.3  -1594.0 | 6.734  47.70 | 1  1 | **0.009**  **<0.001** |
|  |  | -1594.0 | 0.000 | 1 | 0.994 |
|  | *Random effects*  *Vials/block* | *Std Dev* |  |  |  |
|  |  | *4.1* |  |  |  |
| *Rel^E20^* | Sex  Treatment  Sex × Treatment | -1497.2  -1497.3 | 0.048  3.56 | 1  1 | 0.82  0.058 |
|  |  | -1497.1 | 0.20 | 1 | 0.65 |
|  | *Random effects*  *Vials/block* | *Std Dev* |  |  |  |
|  |  | *0.26* |  |  |  |
| *𝝙 AMPs* | Sex  Treatment  Sex × Treatment | -1605.6  -1605.7 | 0.263  1.920 | 1  1 | 0.60  0.16 |
|  |  | -1604.9 | 1.451 | 1 | 0.22 |
|  | *Random effects*  *Vials/block* | *Std Dev* |  |  |  |
|  |  | *0.21* |  |  |  |
| *Group-B* | Sex  Treatment  Sex × Treatment | -1537.3  -1538.8 | 2.950  0.299 | 1  1 | 0.08  0.58 |
|  |  | -1536.9 | 0.771 | 1 | 0.37 |
|  | *Random effects*  *Vials/block* | *Std Dev* |  |  |  |
|  |  | *0.02* |  |  |  |
| *Dpt* | Sex  Treatment  Sex × Treatment | -3386.1  -3398.4 | 24.49  3.157 | 1  1 | **<0.001**  0.08 |
|  |  | -3386.1 | 0.105 | 1 | 0.74 |
|  | *Random effects*  *Vials/block* | *Std Dev* |  |  |  |
|  |  | *0.03* |  |  |  |
| *𝝙 AMPs^+Dpt^* | Sex  Treatment  Sex × Treatment | -2596.1  -2613.2 | 34.22 23.92 | 1  1 | **<0.001**  **<0.001** |
|  |  | -2580.5 | 31.10 | 1 | **<0.001** |
|  | *Random effects*  *Vials/block* | *Std Dev* |  |  |  |
|  |  | *3.73* |  |  |  |
| *Spz* | Sex  Treatment  Sex × Treatment | -1755.6  -1763.0 | 14.86 21.61 | 1  1 | **<0.001**  **<0.001** |
|  |  | -1755.4 | 0.298 | 1 | 0.58 |
|  | *Random effects*  *Vials/block* | *Std Dev* |  |  |  |
|  |  | *0.08* |  |  |  |
