# Supplementary material for: IMD-mediated innate immune priming increases Drosophila survival and reduces pathogen transmission
Source: PLoS Pathog. 2024 Jun 10;20(6):e1012308. doi: 10.1371/journal.ppat.1012308 (PMC11192365; doi:10.1371/journal.ppat.1012308)
Supplement: S11 Table — (DOCX) [file ppat.1012308.s017.docx]

S11 Table. Summary of log10 transformed bacterial load data in AMP deletion lines after 0.2 OD *P. rettgeri* infection, analysed using non-parametric ANOVA (K-W test) by fitting ‘treatment’ (i.e., primed and unprimed) as categorical fixed-effects for male and females of each fly lines (control *w^1118^* and transgenic flies).

| **Fly line** | **Sex** | **Chi Sq.** | **Df** | **P** |
| --- | --- | --- | --- | --- |
| *w^1118^* | *Female* | 1.1165 | 1 | 0.29 |
|  | *Male* | 7.5172 | 1 | **0.006** |
| *Rel^E20^* | *Female*  *Male* | 4.0368  0.2516 | 1  1 | **0.044**  0.61 |
| *Spz* | *Female*  *Male* | 0.0152  0.0050 | 1  1 | 0.90  0.94 |
| *𝝙 AMPs* | *Female*  *Male* | 4.9868  0.3014 | 1  1 | **0.025**  0.58 |
| *Group-B* | *Female*  *Male* | 0.5612  3.5402 | 1  1 | 0.45  0.059 |
| *Dpt* | *Female*  *Male* | 0.2848  0.0020 | 1  1 | 0.59  0.96 |
| *𝝙 AMPs^+Dpt^* | *Female*  *Male* | 0.1589  11.2941 | 1  1 | 0.69  **0.0008** |
