# Supplementary material for: IMD-mediated innate immune priming increases Drosophila survival and reduces pathogen transmission
Source: PLoS Pathog. 2024 Jun 10;20(6):e1012308. doi: 10.1371/journal.ppat.1012308 (PMC11192365; doi:10.1371/journal.ppat.1012308)
Supplement: S12 Table — (DOCX) [file ppat.1012308.s018.docx]

S12 Table. Summary of mixed effects Cox prop-hazard in tissue-specific Dpt knockdown lines, fitting the model to estimate strength of priming response in male and female control w^1118^ and UAS^-RNAi^ tissue-specific mutants. We used data from the unprimed-infected and the primed-infected treatments and specified the model as: survival ~ Treatment x sex, with treatment and sex as fixed effects for each fly line. The table shows model output (ANOVA).

| **Fly strain** | **Source** | **χ2** | **Df** | **P** |
| --- | --- | --- | --- | --- |
| *w^1118-iso^* | Sex  Treatment  Sex × Treatment | 2.306 | 1 | 0.12 |
|  |  | 11.95  3.226 | 1  1 | **0.005**  0.075 |
| *FB>DptB* | Sex  Treatment  Sex × Treatment | 1.996 | 1 | 0.15 |
|  |  | 0.977  0.068 | 1  1 | 0.32  0.79 |
| *HH>DptB* | Sex  Treatment  Sex × Treatment | 3.609 | 1 | 0.057 |
|  |  | 17.06  0.602 | 1  1 | **<0.001**  0.43 |
