# Supplementary material for: IMD-mediated innate immune priming increases Drosophila survival and reduces pathogen transmission
Source: PLoS Pathog. 2024 Jun 10;20(6):e1012308. doi: 10.1371/journal.ppat.1012308 (PMC11192365; doi:10.1371/journal.ppat.1012308)
Supplement: S13 Table — (DOCX) [file ppat.1012308.s019.docx]

S13 Table. Summary of log10 transformed Dpt gene expression data in control w^1118^ flies after 0.2 OD *P. rettgeri* priming and challenge, analysed using ANOVA by fitting ‘treatment’ and ‘sex’ as categorical fixed-effects.

| **Condition** | **Source** | **F value** | **Df** | **P** |
| --- | --- | --- | --- | --- |
| Before secondary exposure (***Dpt***) | Sex  Treatment  Sex × Treatment | 36.96  5.295  0.297 | 1  1  1 | **<0.001**  **<0.001**  0.13 |
| After secondary exposure (***Dpt***) | Sex  Treatment  Sex × Treatment | 0.487  29.48  2.465 | 1  1  1 | 0.49  **<0.001**  0.13 |
| ***AttC*** and ***Dro*** AMP genes expression after secondary exposure | AMP genes  Treatment  Timepoint  Treatment x genes | 98.53  1.704  29.51  0.725 | 1  1  1  1 | **<0.001**  0.20  **<0.001**  0.40 |
|  | Treatment x timepoint | 0.028 | 1 | 0.86 |
|  | Gene x timepoint | 60.07 | 1 | **<0.001** |
|  | Treatment x gene x timepoint | 0.323 | 1 | 0.57 |
