# Supplementary material for: IMD-mediated innate immune priming increases Drosophila survival and reduces pathogen transmission
Source: PLoS Pathog. 2024 Jun 10;20(6):e1012308. doi: 10.1371/journal.ppat.1012308 (PMC11192365; doi:10.1371/journal.ppat.1012308)
Supplement: S14 Table — (DOCX) [file ppat.1012308.s020.docx]

S14 Table. Summary of mixed effects Cox model, fitting the model to estimate priming response in male and female PGRP mutant flies. We used data from the unprimed-infected and the primed-infected treatments and specified the model as: survival ~ Treatment x sex x (1|vial), with treatment and sex as fixed effects, and vials within a block as a random effect for each fly line. The table shows model output (ANOVA).

| **Fly strain** | **Source** | **loglik** | **χ2** | **Df** | **P** |
| --- | --- | --- | --- | --- | --- |
| *PGRP-LB* | Treatment  Sex  Sex × Treatment | -818.79  -818.13 | -0.0048  1.3085 | 1  1 | 1.00  0.25 |
|  |  | -817.98 | 0.3047 | 1 | 0.58 |
|  | *Random effects*  *Vials/block* | *Std Dev* |  |  |  |
|  |  | *9.011949e-03* |  |  |  |
| *PGRP-LC* | Treatment  Sex  Sex × Treatment | -891.65  -891.65 | 0.0189  0.0057 | 1  1 | 0.89  0.96 |
|  |  | -891.64 | 0.0068 | 1 | 0.93 |
|  | *Random effects*  *Vials/block* | *Std Dev* |  |  |  |
|  |  | *9.281555e-03* |  |  |  |
| *PGRP-LE* | Treatment  Sex  Sex × Treatment | -928.63  -928.31 | 0.2609  0.6362 | 1  1 | 0.60  0.42 |
|  |  | -928.31 | 0.0036 | 1 | 0.95 |
|  | *Random effects*  *Vials/block* | *Std Dev* |  |  |  |
|  |  | *0.17* |  |  |  |
