# Supplementary material for: IMD-mediated innate immune priming increases Drosophila survival and reduces pathogen transmission
Source: PLoS Pathog. 2024 Jun 10;20(6):e1012308. doi: 10.1371/journal.ppat.1012308 (PMC11192365; doi:10.1371/journal.ppat.1012308)
Supplement: S15 Table — (DOCX) [file ppat.1012308.s021.docx]

S15 Table. Summary of log10 transformed bacterial load data after 0.2 OD P. rettgeri infection, analysed using non-parametric ANOVA (Kruskal-Wallis test) by fitting ‘treatment’ (i.e., primed and unprimed) as categorical fixed-effects for male and females of PGRP mutants.

| **Fly line** | **Sex** | **Chi Sq.** | **Df** | **P** |
| --- | --- | --- | --- | --- |
| *PGRP-LB* | *Female* | 0.4300 | 1 | 0.51 |
|  | *Male* | 0.3295 | 1 | 0.56 |
| *PGRP-LC* | *Female*  *Male* | 0.6063  0.2020 | 1  1 | 0.43  0.65 |
| *PGRP-LE* | *Female*  *Male* | 0.0643  0.0813 | 1  1 | 0.79  0.77 |
