# Supplementary material for: IMD-mediated innate immune priming increases Drosophila survival and reduces pathogen transmission
Source: PLoS Pathog. 2024 Jun 10;20(6):e1012308. doi: 10.1371/journal.ppat.1012308 (PMC11192365; doi:10.1371/journal.ppat.1012308)
Supplement: S16 Table — (DOCX) [file ppat.1012308.s022.docx]

S16 Table. Summary of log10 transformed Dpt gene expression data in flies with different PGRP deletion transgenic flies after 0.2 OD *P. rettgeri* priming and challenge, analysed using ANOVA by fitting ‘fly line’ (*PGRP-LB, -LC & -LE*) ‘treatment’ (*primed, unprimed*) and ‘sex’ as categorical fixed-effects

| **Source** | **df** | **Sum of Sq.** | **F ratio** | **p** |
| --- | --- | --- | --- | --- |
| Fly line | 2 | 596443.9 | 582.0 | **<.0001** |
| Sex | 1 | 2981.64 | 5.819 | **0.02** |
| Fly line x sex | 2 | 5623.86 | 5.487 | **0.007** |
| Treatment | 1 | 783.67 | 1.529 | 0.22 |
| Fly line x treatment | 2 | 1754.66 | 1.712 | 0.19 |
| Sex x treatment | 1 | 267.07 | 0.521 | 0.47 |
| Fly line sex x treat | 2 | 567.95 | 0.554 | 0.57 |
