# Supplementary material for: IMD-mediated innate immune priming increases Drosophila survival and reduces pathogen transmission
Source: PLoS Pathog. 2024 Jun 10;20(6):e1012308. doi: 10.1371/journal.ppat.1012308 (PMC11192365; doi:10.1371/journal.ppat.1012308)
Supplement: Attachment — Submitted filename: PPATHOGENS-response to reviewers.docx [file ppat.1012308.s023.docx]

**Dear Editorial Board of PLOS Pathogens,**

Thank you for your positive handling of our submission and for giving us the opportunity to revise and resubmit our work to PLOS Pathogens.

We were very pleased to see that both reviewers – while raising helpful (but minor) points for improvement – were overall very positive and even broadly complimentary about the motivation, scope, and execution of our work.

Below we have offered detailed responses and made changes in response to all the reviewers’ comments. All comments were quite straightforward to address. The major change to highlight is that we have taken the advice of Reviewer 2 to remove some of the less central results to supplementary material (the locomotor activity; and we have also moved the *Dpt* expression for PGRP mutants to supplementary material). In addition, we agree with the reviewer that our specificity experiment lacked the necessary controls to allow making any definitive conclusions about the specificity of the priming response, and therefore we agree it was best to remove it from the manuscript.

All other comments were addressed, two figures (Fig 2 and Fig 9) have been replotted to show the raw data points, and further discussion of specific points is now provided, as detailed below.

We hope that you are satisfied with the extent of our revisions, which have resulted in an improved manuscript.

Sincerely,

Pedro Vale (on behalf of all co-authors)

**Editor comments:**

Both reviewers found this to be **an important topic** and the results were **novel** and **interesting**. As well as a number of changes to the text, reviewer 2 questions whether some controls are missing and suggests removing these experiments. This needs careful attention in the revised version. They also suggest that the manuscript is generally simplified, which may increase its impact.


Reviewer's Responses to Questions

**Part I - Summary**
Please use this section to discuss strengths/weaknesses of study, novelty/significance, general execution and scholarship.

Reviewer #1: This study aims to clarify the phenomena of immune priming in invertebrates, which in my opinion is of big importance for several fields (e.g., epidemiology, conservation) and has been heavily studied but in a rather descriptive manner. The authors instead tackled the mechanistic part of the phenomena - very poorly studied in past studies - which I appreciated and **think it contributes substantially to the field**. To my knowledge, there are only a couple of old studies that demonstrate immune priming in Drosophila with bacterial infections. Wolbachia-presence/absence in these studies is also often neglected, and that is not the case here, which I again appreciated. So, it is quite interesting, **and might I dare to say "novel**", that this phenomenon has been found most recently in Drosophila and has been fairly well described in response to this particular bacterial infection. **I would also like to add the importance of the standpoint the authors take in this study**: acknowledging the complexity of infection and how the latter influences shedding and locomotor activity (i.e., potential proxy for contact rate) and therefore, pathogen transmission, which in turn **is crucial and not often observed in the field**. I believe this is rather relevant nowadays and contributes to the understanding of disease evolution and spread in other model systems besides the fruit fly and is well emphasized in the last paragraph of the discussion.
The manuscript is quite well-written, straightforward and easy to follow (**in particular the results were a delight to read** even when they are this dense). The experiments are well design and also quite easy to follow, making a rather complete story in the end. The authors not only observed, expanded, tested for specificity and duration of the immune priming, but also measured its consequences for transmission and the mechanisms underpinning the phenomena with good methodologies. They also were clear about their intentions and the limitations of such methodologies (for example how phagocytosis and melanisation might also play a role). The methods are fairly well-described with the standard nomenclature and **I do not think additional work is necessary and would actually harm the study by undermining some results**.
**I think this study is of great importance for the field** and for the readers of PLOS Pathogens. Moreover, it aligns with the journal interests, and so, I would mostly the authors to address the **overall minor comments** to the manuscript.

**We thank the reviewer for their extensive and overwhelmingly positive comments about the motivation and execution of our work. Below we address all of the minor comments.**

Reviewer #2: This manuscript summarizes many different aspects of immune priming in Drosophila melanogaster against the pathogenic Gram-negative bacterium Providencia rettgeri. The authors characterize the lifespan of the immune priming, its sex and pathogen specificity as well as septic and oral routes of exposure, before investigating more closely the role of different immune pathways and antimicrobial peptides in mounting a successful priming response. They demonstrate that the IMD pathway and the AMP Diptericin are indispensable for successful immune priming against this bacterial pathogen. Finally, they report on transmission rates being influenced by the immune history of an individual and conclude how immune priming can have profound epidemiological consequences.
This study presents **a very thorough investigation** of many diverse aspects of immune priming, which is **a great contribution to the field and broadens our understanding of immune memory and its consequences**. However, I could not help but notice a general lack of focus. **Extremely important and novel findings** are hidden between other experiments that seem rather superficial or not relevant to the story the authors want to tell. This was quite distracting while reading and a more compact version might be more appropriate.

**Thank you for the very positive endorsement of our work. We appreciate the comments regarding the lack of focus. Below we have taken all the reviewer’s comments on board to increase the clarity of our writing and of the study.**

**Part II – Major Issues: Key Experiments Required for Acceptance**
Please use this section to detail the key new experiments or modifications of existing experiments that should be absolutely required to validate study conclusions.

Generally, there should be no more than 3 such required experiments or major modifications for a "Major Revision" recommendation. If more than 3 experiments are necessary to validate the study conclusions, then you are encouraged to recommend "Reject".

Reviewer #1: I think the discussion could benefit of a bit more explaining, although I understand the difficulty in doing so with this number of results. However, here are some examples:

**We have expanded the discussion following the points below.**

• sexual dimorphism in fruit flies has been associated to differences in ROS expression in the gut and other tissues, what might explain in part of the sexual dimorphic results we observe here.

**Thank you for this suggestion. It’s difficult to know where the sexual dimorphism in priming arises from – just as sex differences in immunity more generally seem to have multiple causes (Toll, AMPs, trade-offs, etc) as we mention in the discussion. We imagine the reviewer is referring specifically to the lack of priming in female flies during oral infection (Fig 4). Differences in ROS might explain this example of sexual dimorphism, but would not explain why we did not see it during the septic infection in the same figure (despite seeing it in multiple other experiments (Figs 1, 2, 3, 6). It also does not explain why the effect is so long lasting in males, but rather short-lived in females in Fig 1. In any case, we completely agree that this requires more in-depth discussion, and we have now expanded on these points L682 onwards.**

• Wolbachia is a gram-negative bacterium but might still induce different immune effectors, that trade-off with the ones needed for Providencia.

**This is an interesting point, which we had not fully considered. It’s not clear that co-infection with Wol and P. *rettgeri* would generate a trade-off between the expression of different AMPs, but it certainly an idea worth speculating about. We have added a sentence to this effect on L728-730.**

• The lack of locomotor activity could be better discussed and, in my opinion, might reflect the transmission route (which is briefly mentioned in the results) of the pathogen, that seems to take a more sit and wait approach.

**Behavioural changes in response to infection are often difficult to interpret – they may be the outcome of resource reallocation, or of pathology, and therefore sometimes we see these changes in locomotor activity ,while other times the effects are more subtle. In this case, we simply wanted to test if the difference we saw in the transmission assays could be ascribed mainly to the effect of priming on shedding (and not some behavioural competence of transmission). The lack of effect on activity confirmed that effects on shedding were the main driver of differences in transmission. In light of other comments from Reviewer 2, we have also decided to move this figure to supplementary material, as to not distract from the main focus of the paper.**

• In the transmission experiment, you exposed them for 4 hours to the infected fly and then immediately homogenised the individuals. I wonder if that time is enough to measure transmission and if maybe you should have individualized the recipient flies, waited a bit longer and then measured the presence/absence of transmission. Particularly because I am not aware of your CFU detection threshold.

**This is an excellent observation. Our choice of time-period was informed by a lot of work we’ve been doing to develop a robust transmission assay (there is another quite substantial manuscript about his in prep). We’ve found that once infected via the oral route, flies shed the pathogen substantially in the first 4-6 hours and shedding then decreases until 8-12 h post-infection. Therefore, during the first 4 hours our donor fly was shedding substantially and our initially uninfected recipient flies were being exposed to high amounts of shed pathogen (10e4 to 10e6 CFU per fly!). The problem arises because we want to measure the transmission caused directly by the focal donor fly, and not transmission caused by the recipient flies once they become infected. We therefore decided to wait four hours to allow enough time for most shedding to occur, but not long enough that new infections could be spread. The reviewer raises a good point about housing the flies individually after exposure – this is something we will definitely implement and test. However, even without this period to allow infections to grow, we still detected infection in 75% of recipient flies (Fig 5B)**

Reviewer #2: -

**Part III – Minor Issues: Editorial and Data Presentation Modifications**
Please use this section for editorial suggestions as well as relatively minor modifications of existing data that would enhance clarity.

Reviewer #1: Minor comments:
• Given that the results come before the methods, I would suggest paying attention to some important nuances of the methods, like the infection route used in some of the experiments, which is only explicitly mentioned later on.

**Thank you for this suggestion – the infection route was indeed unclear. We have added additional detail to all the results where we now specify if each infection was systemic or orally acquired.**

• References are not consistently formatted throughout the manuscript. For instance, introduction is numeric, but in line 154 is not.

**Thank you for noticing this lapse, we corrected the references, but note that this section has since been removed in light of comments from reviewer 2.**

Reviewer #2: I would recommend streamlining the manuscript by removing some experiments that are lacking controls and by reducing the number of figures.

**Thank you for your suggestions to improve and streamline the paper.**

The experiment on the species specificity does not consider the potential differences in virulence between the different bacteria taxa. To ensure that the priming is really species specific the priming-infection set up should have been fully reciprocal, also using the other bacteria for the priming. Or at least the exposure of an unprimed control group should have been tested with the other bacteria. Only the difference in survival between an unprimed and primed group will let us estimate a priming benefit. (Here, also the hazard ratios in Figure 3 B and C are shown twice, the only difference being the chosen reference.)

**The reviewer raises some very valid points, and clearly it is difficult to make strong conclusions about the specificity of the response without a fully reciprocal design. Considering this, we agree the best is to remove this experiment/figure and to remove any mention of specificity throughout the manuscript.**

The experiment regarding the locomotor activity is currently only described very briefly in the text and the different aspects of Figure 7 are not discussed in any detail. Thus, this figure might be more suited for the supplement.

**We agree this result is not central to the paper, and we have moved it to the supplement, as suggested by the reviewer.**

My concern in the experiments using the AMP mutants (Figure 9 Ai-iii) is that these have a really high susceptibility to infection with almost all flies dying, which makes it a lot more difficult to detect a priming effect. On the other hand, the finding that the priming effect can be rescued by the expression of Diptericin in the males but not females is highly interesting and should be given more importance. The same is true for the tissue specific Diptericin knockdowns with their quite exciting results (which also tend to get lost in this long list of separate experiments).

**We understand the concern with the AMP mutants having high susceptibility, however this was expected, and helped us to narrow down the priming phenotype to requiring just a single AMP. See also our responses below to a similar question regarding the Relish mutant. The rationale was always to start quite bluntly (deleting all AMP -related IMD-mediated immunity) and then narrowing down to more specific AMP responses. The tissue-specific results are an extension of this rationale – once we knew it was Dpt, it made sense to ask where it was important. We hope that now that we have streamlined the manuscript, this line of thought is now clearer and reflect the importance of these results.**

Lastly, the authors describe that their use Cox mixed effect models testing not only for the effect of priming treatment but also for differences between the sexes, however the results of the tests for sex differences are not discussed

**Apologies for such a important omission, especially given how often we discuss sex differences. We have now added explicit mention of sex effects on L131-133, L162-172, L213-214, L382, L503**

Minor points:
The intro could mention more examples of priming from other insects/invertebrates (besides Drosophila and woodlouse)

**The paragraph focusing on Drosophila and woodlouse is biased toward studies where priming mechanisms have been elucidated in some detail – this is not common for many other insects/invertebrates. However, we have now added a section to the end of the previous paragraph that details examples of priming in many other insect/invertebrate orders.**

Figure 1: why is there no challenge control (heat killed bacteria) in the later time points

**This was due to an unfortunate miscalculation. All time-point treatments (Fig 1A-E) were originally set up simultaneously, and all received either an initial exposure to heat-killed *P. rettgeri* (green) or PBS (grey). Then, at each time-point that we wished to test a priming effect, a subset of the heat-killed treated flies were given a secondary challenge (red). Partly due a shortage of fly numbers, and partly because we did not expect the priming effect to be so long-lasting, we ended up using all the HK-only flies (green) in order to expose them to a secondary challenge at later time-points (red).**

**However, we did this in the knowledge that even at long exposure times (3-4 weeks) flies initially exposed to a HK challenge experienced extremely low mortality (under 5%). Below we plot data using the same w1118 line confirming this – this was recorded in a separate experiment and therefore not included in these plots – but confirming that HK treatment early on does not cause substantial mortality on the timescale studied here (2 weeks in Figure 1).**

How strong is the effect of the vials (depending on how sick individuals become might affect the rest of the cohort)?

**Table S1 summarises our linear mixed models, where vial(and block) was included as a random effect and shows that the proportion of variance explained by vial is very low, almost negligible.**

Figure 2: Is Fig2a the same as Fig1c?

**That’s correct – apologies, we should have made this explicit in the figure legend. The aim of the experiments in Fig 2 was to test the generality of the priming effect. Given we had already measured the effect in w1118 in Fig 1, and that we focused on 96h following the initial exposure, we thought it would be helpful to include that panel here to allow a more direct comparison with the other lines, within the same figure. We now make this clear in the figure legend.**

L141 How the labels are referenced in the text does not seem correct. 2C should be 2D, l140 2B should be 2 C

**Thank you for picking up on this error. We have corrected all references to the figures in the main text.**

Figure 5: There is also no priming via the septic route in the females. However, this was observed in previous experiments (figure 1). Thus, the results for the females don’t seem repeatable here and should not be attributed to the priming/infection route. The authors should either repeat this experiment or discuss the lack of septic priming in the females in this part of the study.

**This is indeed a puzzling and frustrating outcome. In terms of repeating the experiment, we have indeed repeated it several times throughout the manuscript – Fig 1, 3, 6 are all independent experiments, all showing a clear benefit of priming in females. So there is no doubt that it can happen in females. Why sometimes it does not, remains puzzling to us. When seeing this result in Fig 4, it would be tempting to assume something had gone wrong, and omit these results – after all we have several experiments showing it happens in females. However, it is notable that we also do not recover a priming benefit in females when restoring the function of Dtp – only in males. It therefore became clear to us that for some unknown reason, priming in females is more variable than in males. We thought it was important to show this, even it makes the story a little less clear!**

**These aspects were not adequately discussed in the previous version, and we now expand on this in the discussion (L692 onwards).**

Figure 6B: Would it be possible to add individual data points here?

**This is a sensible suggestion, we have re-plotted the figure to show all data points.**

Figure 7: Is barely mentioned in the text. If the results have this little importance, the figure should be moved to the supplements.

**We have now moved this to supplementary material (Fig S3).**

Figure 8: The Relish mutant seems to have no resistance at all. So it would be rather difficult to detect a priming effect if in an unprimed state no flies survive the infection.

**Yes, but this was expected. Here we were taking a rather blunt approach simply to test if priming was regulated in any way by the toll pathway (it was not) which then allowed us to probe more specific IMD-mediated AMP responses. We would never get a subtle phenotype from a Relish mutant, but it pointed us in the right direction.**

Figure11 A): the dots should not be connected as they are not repeated measures of the same fly. Also, it is not clear what the reference is? Is it the mean of the three time points for uninfected flies?

**The reviewer is correct. We have replotted the figure, removing the connecting lines, but also replacing the large dots (which showed the mean expression) with boxplots and raw data points for each timepoint.**

**Regarding the calculation of the expression relative to uninfected controls, this was done using standard approaches where the relative fold-change difference in expression between infected and health control flies was calculated as described in Livak and Schmittegen 2001. Briefly:**

**Fold change = 2^-ΔΔCt^**

**Where, ΔΔCt = [(Ct of Gene A – Ct of Internal control) of Infected sample] –**

**[(Ct of Gene A – Ct of Internal control) of Control sample]**

**Livak KJ, Schmittgen TD. 2001 Analysis of relative gene expression data using real-‐‑time quantitative PCR and the 2(-‐‑Delta Delta C(T)) Method. Methods San Diego Calif 25, 402–408. (doi:10.1006/meth.2001.1262)**

**However, we realise this was not clearly described in the methods, and we have now added these important details to L819.**

Line 393 the wrong figure is cited here, while the actual figure 12 is not introduced in the text before its appearance.

**We have now corrected the figure referencing throughout, and moved the figure after it is first referenced (now Fig 10).**
